# Preparation of novel Zn–Al layered double hydroxide composite as adsorbent for removal of organophosphorus insecticides from water

**DOI:** 10.1038/s41598-023-37070-8

**Published:** 2023-06-23

**Authors:** Nastaran Ghanbari, Hossein Ghafuri

**Affiliations:** Catalysts and Organic Synthesis Research Laboratory, Department of Chemistry, Iran University of Science and Technology, Tehran, 16846‑13114 Iran

**Keywords:** Environmental sciences, Environmental social sciences

## Abstract

In this work, a new and efficient composite LDH with high adsorption power using layered double hydroxide (LDH), 2,4-toluene diisocyanate (TDI), and tris (hydroxymethyl) aminomethane (THAM) was designed and prepared, which was used as an adsorbent to adsorb diazinon from contaminated water. The chemical composition and morphology of the adsorbent were evaluated using Fourier transform infrared (FTIR), X-ray diffraction (XRD), thermal gravimetric analysis (TGA), Energy dispersive X-ray (EDX) and Field emission scanning electron microscopy (FESEM) techniques. Also, the optimal conditions for adsorption of diazinon from water were determined by LDH@TDI@THAM composite. Various parameters like the effect of adsorbent dosage, pH, concentration and contact time of diazinon were studied to determine the optimal adsorption conditions. Then, different isotherm models and kinetic adsorption were used to describe the equilibrium data and kinetic. Also, the maximum adsorption capacity is obtained when the pH of the solution is 7. The maximum adsorption capacity for LDH@TDI@THAM composite was 1000 mg/g at 65 °C and the negative values of ΔG indicate that the adsorption process is spontaneous. After that, studying the reusability of LDH@TDI@THAM composite showed that the removal of diazinon by LDH@TDI@THAM was possible for up to four periods without a significant decrease in performance.

## Introduction

Today, the widespread use of pesticides for pest control and agricultural development, as well as improper wastewater disposal has led to surface and groundwater pollution, hence removal and Adsorption from water resources is very important^1–4^. Chemical pesticides enter surface and groundwater sources through various means such as direct washing of pesticides, sewage disposal, agricultural drainage water, erosion and air, which threatens human health and the environment, thus leading to there has been considerable concern^5–9^.Various techniques that have been studied to remove pesticides from contaminated water include coagulation/flocculation/sedimentation, membrane filtration, adsorption, and advanced oxidation processes, and biodegradation^10–16^. Each of these proposed methods is less considered due to disadvantages such as high investment costs, poor performance and secondary pollution and is used in a limited way in wastewater treatment^17–19^. Hence, absorption has received considerable attention because of simple efficiency, use on a large scale, regenerative capacity, and cost of removing contaminants from water^20,21^. Today, organophosphate pesticides are widely used around the world for pest control due to the ban on the use of organochlorine insecticides^22,23^. Organophosphate compounds are one of the broad and diverse groups of toxic compounds that include pesticides. Organophosphate pesticides are relatively volatile and are very dangerous as neurotoxins to humans and animals. Diazinon with the formulation of O,O-diethyl O-[6-methyl-2-(1-methylethyl)-4-pyrimidinyl] phosphorothioate can be used as an organophosphate insecticide for eliminating flies and mites in a variety of plant and ornamental products in agriculture and home because of its low cost and high efficiency^24–27^. The World Health Organization (WHO) has classified it as a second-class pesticide in terms of toxicity^28–30^. Diazinon is one of the inhibitors of the enzyme acetylcholinesterase. Therefore, its removal from water resources is very important. The highest permissible concentration of this pesticide in drinking water is 0.1 µg/l, which is higher than this limit and has destructive effects on living organisms^31^.

Diazinon can be removed from aqueous solutions by various methods such as biochemical decomposition^32^, membrane separation^33^, oxidation^34^, photocatalysis^35^ and adsorption^36^. Also, Among the various types of methods mentioned, adsorption due to simplicity, biocompatibility and high efficiency as a more appropriate method of removing diazinon from water sources has received much attention. Hence, in the past decades, various adsorbents such as magnetic materials^37^, carbon nanotubes^38^, silica particles^39^, organic porous polymers^40^ and nano-adsorbents^41,42^ have been reported to remove diazinon.

Two-dimensional nanomaterials of layered double hydroxides (LDHs) with sheet-like structures due to their various desirable properties have many applications in various fields^43–50^. LDHs as a new and widely used class of natural or synthetic anionic mineral layered nanomaterials with the formula [M^2+^_(1−x)_ M^3+^_(x)_ (OH)_2_]^x+^ A^n−^_x/n_. yH_2_O where M^2+^ is a divalent metal ion (Zn^2+^, Cu^2+^, Mg^2+^, etc.), M^3+^ is a trivalent metal ion (V^3+^, Ga^3+^, Al^3+^, etc.), and A^n^ is an anion. The charge density of LDH layers (CO_2_^3−^, NO^−^_3,_ Cl^−^) is x = M^3+^/M^2+^  + M^3+^, which is between 0.2 and 0.33 for pure LDH^47,51^.

Adsorbents based on layered double hydroxide (LDH) have provided a new path for the design and preparation of new adsorbents for the degradation of pollutants^52,53^. These adsorbents because of their very good physical and chemical properties, adjustable interlayer distance, high anion exchange capacity, wide light absorption range, special layer structure, ease of synthesis, low cost and recyclability have special place among the adsorbents^54^.

Therefore, in this work, according to the mentioned contents about the importance of removing diazinon insecticide from aqueous solutions, an efficient and reusable adsorbent with good adsorption capacity was designed and prepared. Also, a facile approach is used to fabricate an environmentally-friendly composite adsorbent comprising LDH and organic compounds for the removal of organophosphate insecticide contaminants (e.g. diazinon). The components of this novel composite as absorbent include tris(hydroxymethyl)aminomethane (THAM) grafted on the surface of LDH by organic bridges (2,4-toluene diisocyanate). THAM and 2,4-toluene diisocyanate (TDI) groups placed on the surface and between the LDH layers have increased the adsorption capacity on the LDH surface by increasing the functional groups effective (NH_2_, OH, and aromatic rings) in diazinon absorption. Hence, the composite prepared (LDH@TDI@THAM) here was used as a strong adsorbent with high adsorption capacity to remove diazinon insecticide from aqueous solutions. The performance of the synthesized nanocomposite has been investigated by changing various factors such as dye concentration, removal temperature, pH, adsorbent dose, and adsorbent recyclability. The isotherms and adsorption kinetics of fabricated composite have been also investigated to show the effect of LDH@TDI@THAM composite on the adsorption of diazinon.

## Results and discussion

### LDH@TDI@THAM composite characterization

The successful synthesis of LDH@TDI@THAM composite was confirmed by different techniques such as FTIR, EDX, XRD, FESEM, and TGA.

**Figure 1:**
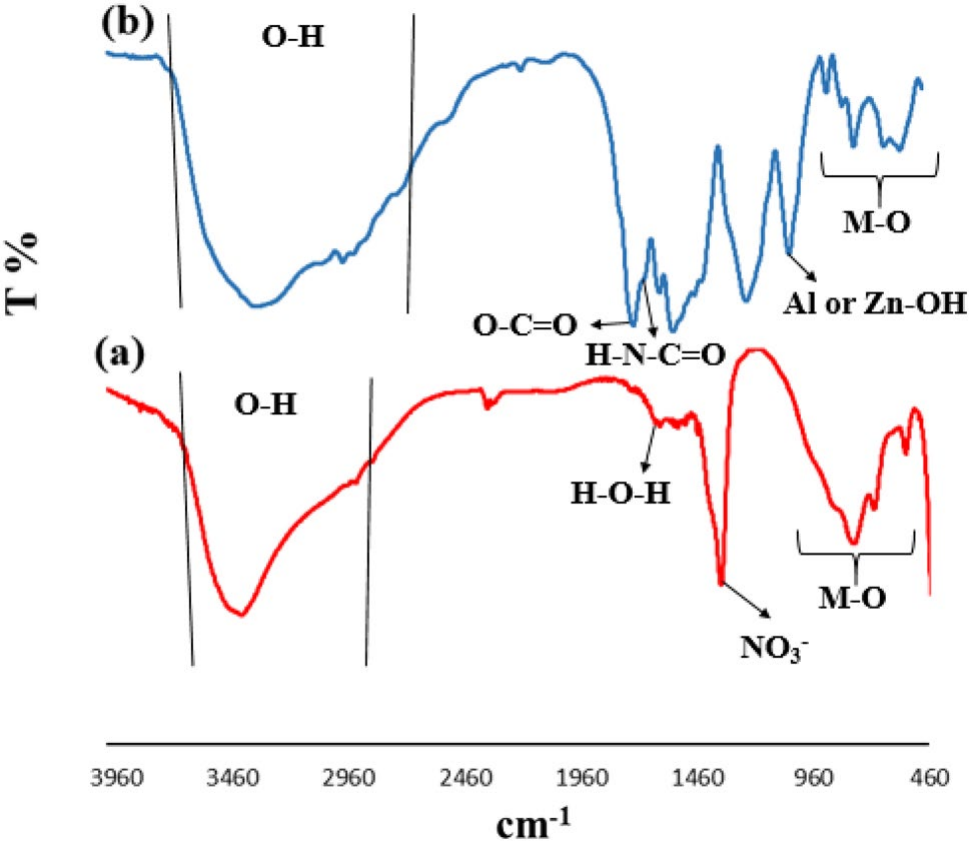
FTIR spectra obtained for (**a**) Zn–Al LDH, and (**b**) LDH@TDI@THAM composite.

Figure 1 shows FTIR spectra obtained for both the Zn–Al LDH and LDH@TDI@THAM composite. The adsorption band at 3425 cm^−1^ related to the stretching vibration of O–H bond (Fig. 1a). The adsorption bands at 1616 and 1356 cm^−1^ are related to the bending vibration of H–O–H in water and interlamellar nitrate anions. The absorption bands between 879 and 475 cm^−1^ are attributed to the vibration of M–O bonds (M can be Al or Zn)^55^.

The FTIR spectrum of LDH@TDI@THAM composite is shown in Fig. 1b. The band at 3328 cm^−1^ corresponds to the vibration of the OH in LDH, and THAM while the band at 2973 cm^−1^ is related to the stretching vibration of C–H bond in THAM. In addition, the adsorption bands at 1735 cm^−1^ and 1654 cm^−1^ can be attributed to the stretching vibration of carbonyl in ester and amide groups in the composite, respectively. The bands between 1036 and 559 cm^−1^ belong to the Metal-O and Al–OH bonds.

**Figure 2:**
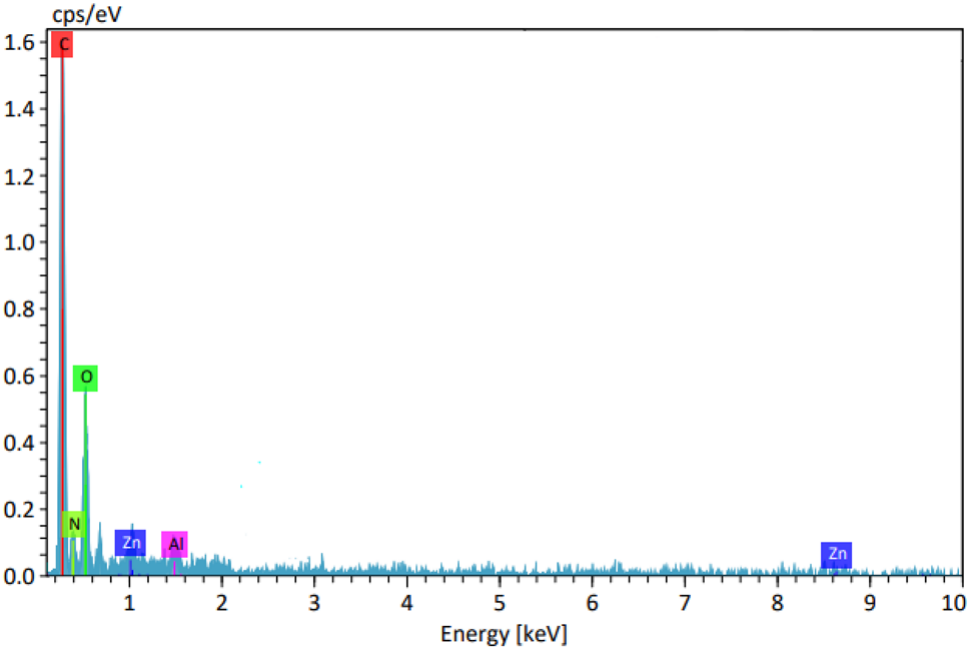
EDX spectra of LDH@TDI@THAM composite.

Figure 2 shows the EDX analysis of LDH@TDI@THAM composite, in which the presence of elements C (52.40%), O (26.39%), N (12.81%), Al (4.28%) and Zn (4.12%) well proves the formation of the composite.

**Figure 3:**
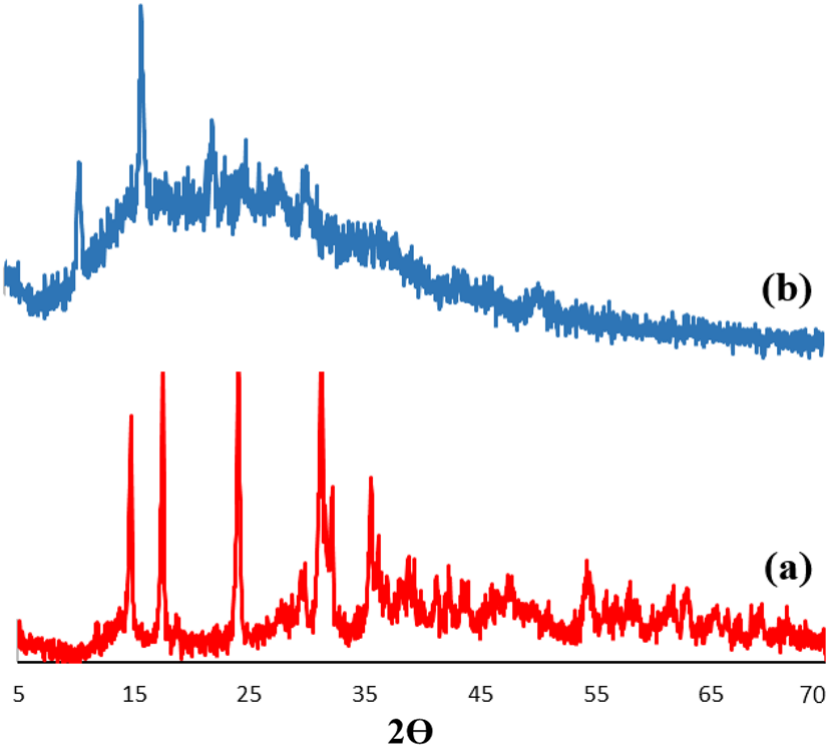
XRD patterns of (**a**) Zn–Al LDH, and (**b**) LDH@TDI@THAM composite.

As can be seen, The XRD patterns of Zn–Al LDH and LDH@TDI@THAM composites are presented in Fig. 3. Also, Fig. 3a, there are symmetrical and sharp reflections at 2θ of 14.80°, 17.55°, 24.04°, 31.25°, 35.59°, 39.30°, 47.75°, 57.99°, and 63.01°, respectively, which determine the structure of Zn–Al LDH^56^.

In addition, The XRD pattern of LDH@TDI@THAM composite is shown in Fig. 3b, which approves the existence of Zn–Al LDH along with other components of the prepared composite. The peaks related to Zn–Al LDH can be clearly seen at 12.80°, 19.14°, 26.47°, 29.98°, 36.27° and 60.54° which is the displacement of the peaks due to its composite with THAM. The well-known amorphous halo at 2θ = 20–30° clearly confirms the amorphous nature of THAM in the synthesized composite.

**Figure 4:**
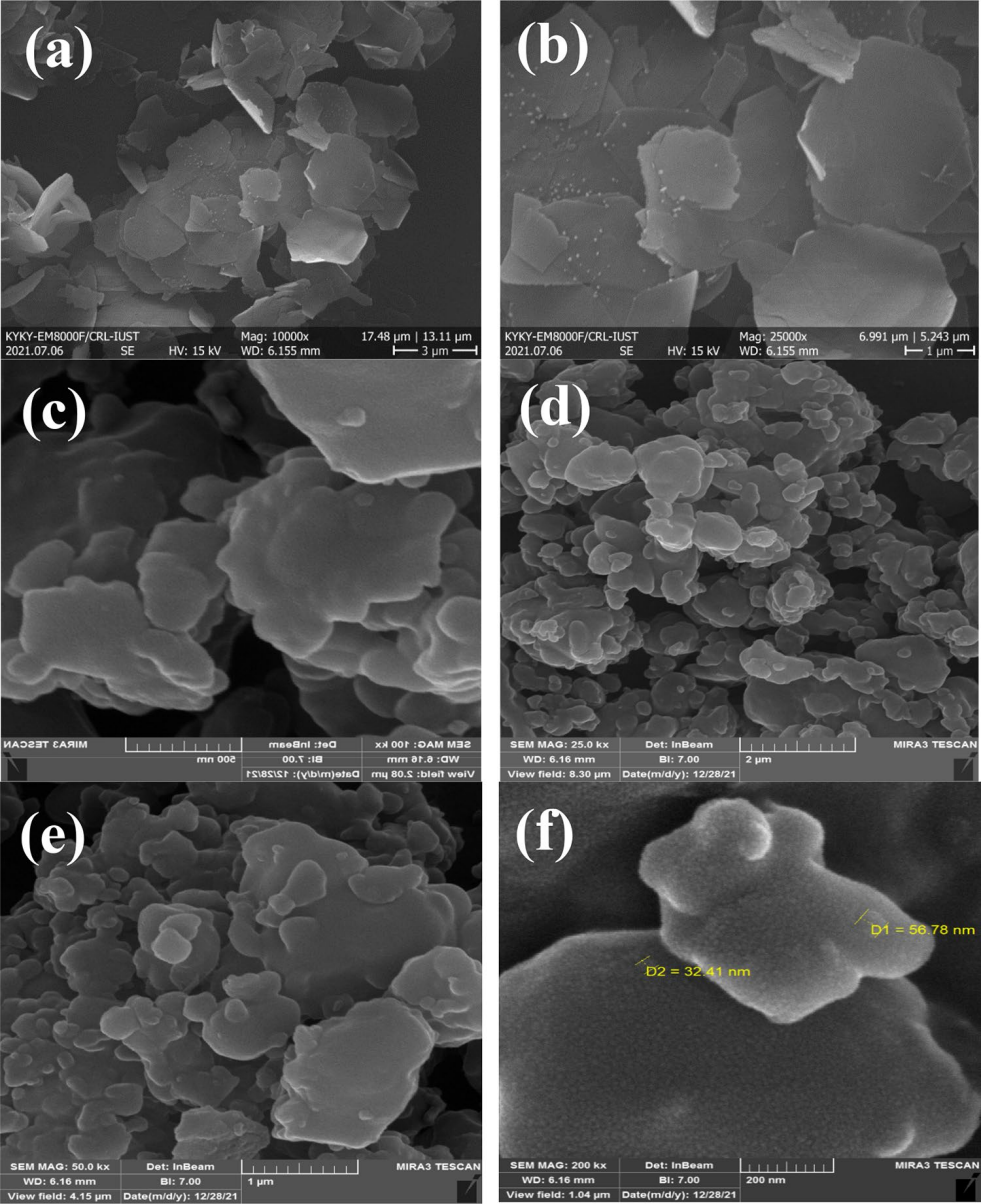
FESEM images of Zn–Al LDH (**a** and **b**) and LDH@TDI@THAM composites (**c**–**f**).

FESEM images of Zn–Al LDH and LDH@TDI@THAM composites are shown in Fig. 4. As can be seen FESEM images Zn–Al LDH shows regular and stacked hexagonal plates (Fig. 4a and b). In addition, images c–f show the morphology of the prepared composite. this images can clearly be seen to increase the diameter of the composite plate compared to raw LDH. Therefore, due to the placement of other components of the composite next to the LDH, the final structure has been created in the form of irregular plates with a larger diameter. This irregularity and increase in diameter can indicate that the composite has been prepared successfully.

**Figure 5:**
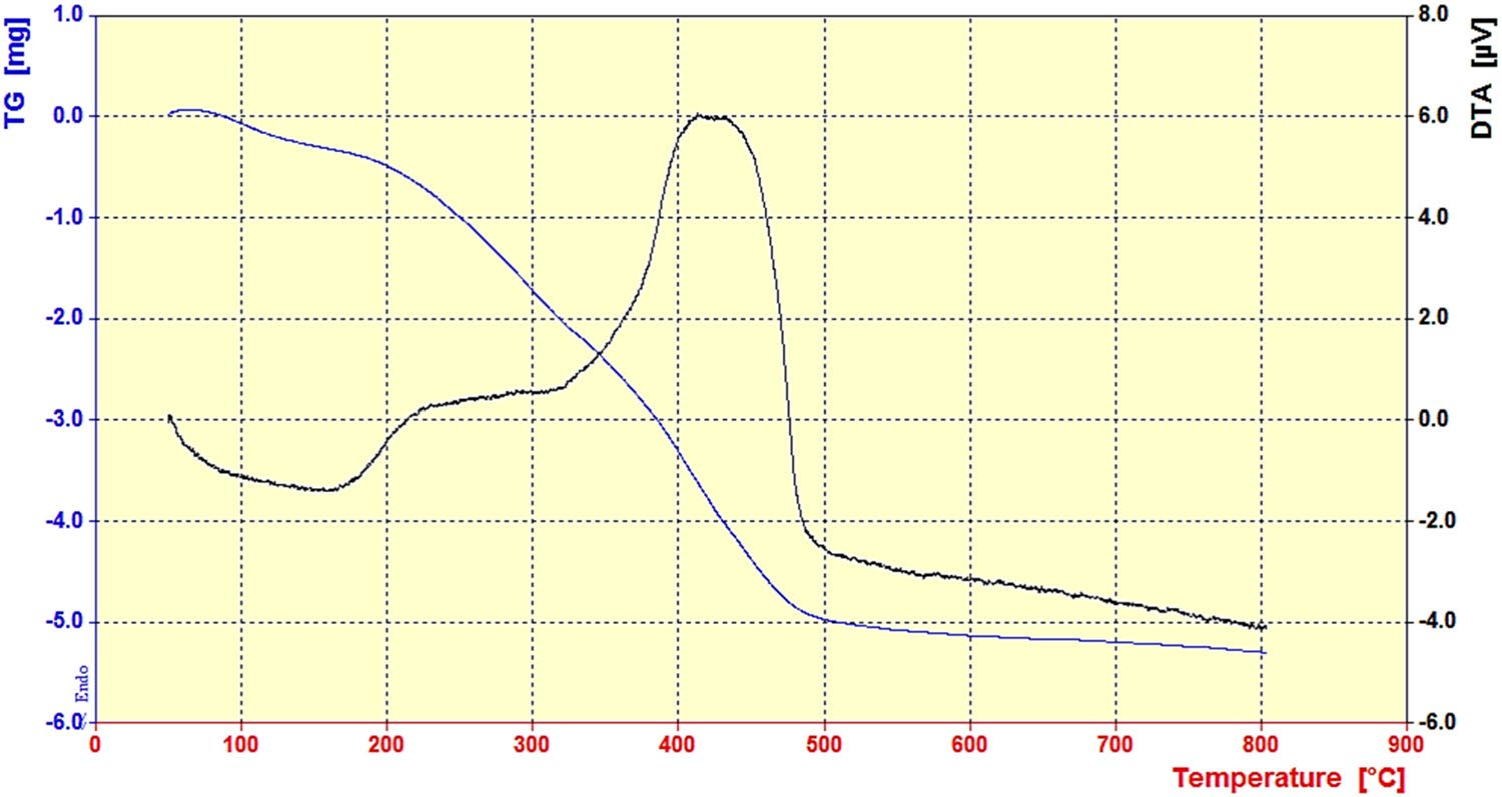
TGA curve of the LDH@TDI@THAM composite.

Using TGA analysis, the thermal stability of LDH@TDI@THAM composite was investigated (Fig. 5). TGA curve shows two weight reductions in the region of 100–150 °C and 200–480 °C, the first decrease is related to the removal of water and solvent molecules absorbed between the layers and the surface of the composite. Also, the second reduction that occurred in the region of 200–480 °C is related to the decomposition of the organic parts of the prepared composite structure. In addition, the curve from the temperature of 480 °C has a constant slope, which is related to the mineral parts (Zn–Al LDH) of the composite.

### Adsorption experiments

Experiments were performed for checking the effect of important adsorption parameters including concentration, adsorbent dose, temperature, time, and pH on diazinon pesticide adsorption in LDH@TDI@THAM composite. After performing the desired tests, filtration was used to remove the adsorbent from the aqueous solution. The maximum absorbance of diazinon was considered as the absorbance value. Diazinon concentration was checked using UV–vis spectrophotometer. The elimination efficiency and the adsorption capacity (q_e_) (mg/g) of diazinon were studied by Eqs. (1) and (2):

1 $$\mathrm{Removal\, \%}=\frac{\left({\mathrm{C}}_{0}- {\mathrm{C}}_{\mathrm{e}}\right)}{{\mathrm{C}}_{0}}\times 100$$

2 $${\mathrm{q}}_{\mathrm{e}}=\frac{\mathrm{V }\left({\mathrm{C}}_{0}- {\mathrm{C}}_{\mathrm{e}}\right)}{\mathrm{W}}$$

diazinon concentration (C_o_ (mg/L)), equilibrium concentration of diazinon (C_e_ (mg/L)) value of sorbent (W (mg)) and volume of diazinon solution (V (L)).

### Effect of concentration

**Figure 6:**
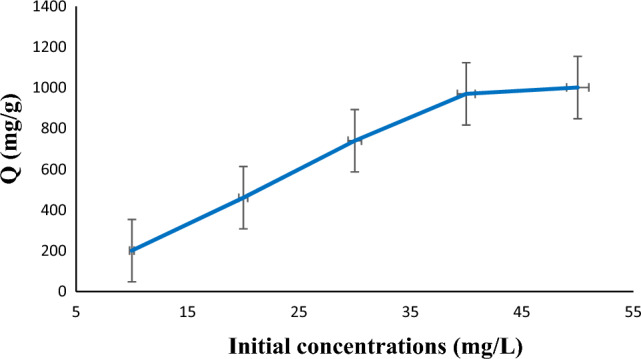
Effect of concentration for the adsorption of diazinon (C_0_ = 10–50 mg/L, pH = 7, T = 25 °C, m = 1 mg, t = 60 min).

Next, to find out the optimal concentration of diazinon, 1 mg of absorbent was added to 25 mL of solution having various concentrations of diazinon (10–50 mg/L) with pH = 7 at 25 °C for 60 min. Afterward, filtration was applied to separate the adsorbent from the solution. The absorption was analyzed by using UV–vis spectrophotometer at wavelength of 247 nm. The obtained values for the maximum absorption showed that the concentration of 40 mg/L can be considered as the optimal concentration for diazinon absorption (Fig. 6).

### Effect of adsorbent dose

**Figure 7:**
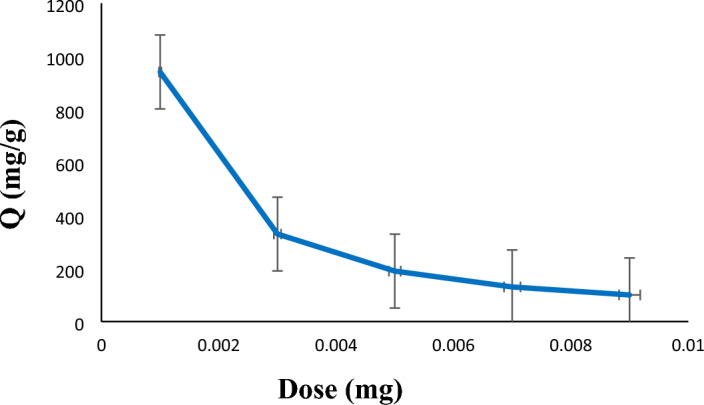
Effect of the amount of the adsorbent on the diazinon adsorption.

Different amounts of the adsorbent (1–9 mg) were used to investigate the absorption of diazinon pesticide in the presence of LDH@TDI@THAM composite. As shown in Fig. 7, the adsorption capacity of diazinon decreases when the amount of the adsorbent increases from 1 to 9 mg. Therefore, this decrease in the adsorption capacity of diazinon by LDH@TDI@THAM composite is due to the accumulation of adsorbent particles, followed by less access to the active sites of the adsorbent. Thus, by increasing the value of LDH@TDI@THAM composite, the number of empty sites available for diazinon absorption, hence, leads to a decrease in diazinon absorption.

### Effect of temperature

**Figure 8:**
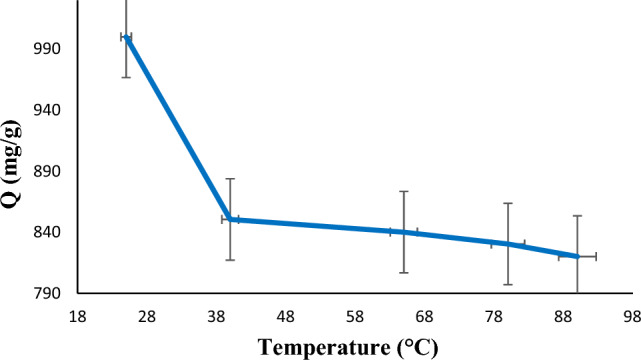
Effect of temperature on the diazinon adsorption.

In various temperatures (25, 40, 65, 80, and 90 °C), the effect of temperature on capacity of Zn–Al LDH composite for diazinon adsorption was investigated (Fig. 8). As can be seen in Fig. 8, adsorption capacity decreases by increasing the temperature from 25 to 40 °C. At temperatures higher than 40 °C, the adsorption capacity further decreases, indicating the exothermic nature of diazinon adsorption on the LDH@TDI@THAM composite adsorbent.

### Effect of time

**Figure 9:**
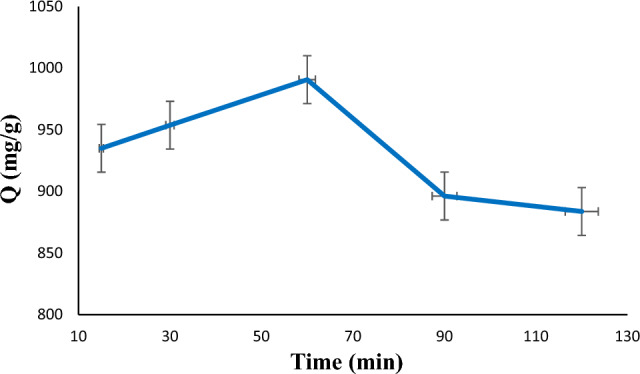
Effect of time on the diazinon adsorption.

Adsorption kinetics of diazinon absorption with an optimal amount of adsorbent (i.e., 1 mg) at pH 7 was investigated at different times (15, 30, 60, 90, and 120 min). Figure 9 shows in equilibrium time (60 min), diazinon has the highest adsorption capacity owing to the presence of more vacancies on the LDH@TDI@THAM composite surface. Also, with the time increases from 60 to 120 min, the adsorption capacity of diazinon decreases. This reduction in absorption is probably due to the formation of a single layer of diazinon on the LDH@TDI@THAM composite surface. In addition, it is possible that this decrease is due to insufficient free space for absorption after equilibrium is reached. It can be seen that the adsorption capacity increases by further increasing the adsorption time from 15 to 60 min. Therefore, 60 min was chosen as the optimal contact time.

### Influence of solution pH

**Figure 10:**
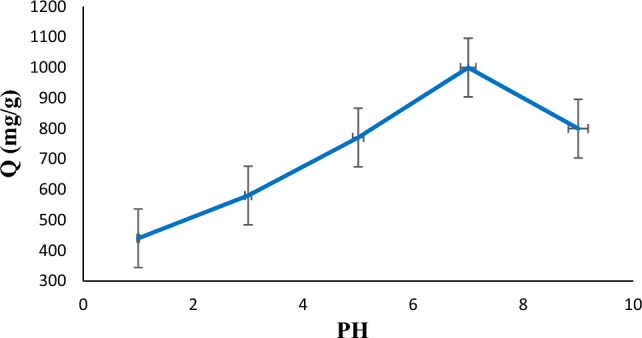
Effect of pH for the adsorption of diazinon (C_0_ = 40 mg/L, pH = 7, T = 25 °C, m = 1 mg, t = 60 min).

Figure 10 shows the dependence of diazinon absorption on the pH of the solution. Diazinon absorption was measured at different pH (1–9) to determine the optimal pH value. As can be seen, the maximum adsorption capacity is obtained when the pH of the solution is 7. Also, decreasing or increasing the pH from 7 will decrease the absorption capacity.

### Kinetics study

**Figure 11:**
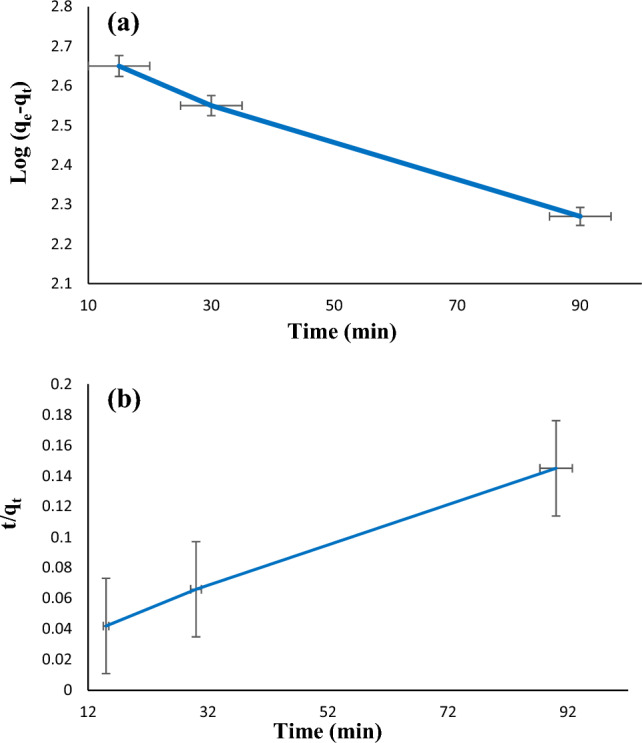
Kinetics of diazinon adsorption by LDH@TDI@THAM composite: pseudo-first-order (**a**), and pseudo-second-order (**b**) models.

Diazinon absorption kinetics was investigated in the presence of 1 mg of adsorbent, concentration 40 mg/L, time 60 min, temperature 40 °C, and pH 7. Stirring of the mixture was performed for 15, 30, 60, 90, and 120 min. In addition, the solutions were filtered to confidence the absence of adsorbents. For analyzing the kinetic data of diazinon adsorption, pseudo-first-order and pseudo-second-order models were applied according to Eqs. (3) and (4), respectively.

3 $$\mathrm{log }({\mathrm{q}}_{\mathrm{e}} - {\mathrm{q}}_{\mathrm{t}})=\mathrm{ log }{\mathrm{q}}_{\mathrm{e}}-\left(\frac{{\mathrm{K}}_{1}}{2.303}\right)\mathrm{t}$$

4 $$\frac{\mathrm{t}}{{\mathrm{q}}_{\mathrm{t}}}=\frac{1}{{\mathrm{K}}_{2 }{\mathrm{q}}_{\mathrm{e}}^{2}}+\left(\frac{1}{{\mathrm{q}}_{\mathrm{e}}}\right)\mathrm{t}$$

where k_1_ (1/min) is the first-order adsorption kinetic, k_2_ (g/mg.min) is the second-order adsorption kinetic, q_t_ (mg/g) is the adsorption capacity, and q_e_ (mg/g) is the adsorption capacity in time. Various kinetic models related to diazinon adsorption are summarized in Fig. 11 and Table 1. The correlation coefficient (R^2^) of pseudo-first-order (a) and pseudo-second-order models (b) for LDH@TDI@THAM composite was 0.9956 and 0.9988, respectively. Therefore, the results show that the adsorption of diazinon on LDH@TDI@THAM composite follows the pseudo-second-order kinetic model.

**Table 1:** Kinetic parameters for diazinon adsorption on LDH@TDI@THAM composite.

Models		LDH@TDI@THAM composite
Pseudo-first-order	q_e_ (mg/g)	70.79
	k	0.02
	R^2^	0.9956
Pseudo-second-order	q_e_ (mg/g)	1000
	k	0.001
	R^2^	0.9988

### Adsorption isotherms

Freundlich (Eq. 5), Langmuir (Eq. 6), and Temkin (Eq. 7) isotherms were also applied for more check the diazinon adsorption on LDH@TDI@THAM composite.

5 $${\mathrm{lnq}}_{\mathrm{e}}={\mathrm{lnK}}_{\mathrm{F}}+\left(\frac{1}{\mathrm{n}}\right){\mathrm{lnC}}_{\mathrm{e}}$$

6 $$\frac{{\mathrm{C}}_{\mathrm{e}}}{{\mathrm{q}}_{\mathrm{e}}}=\frac{1}{{\mathrm{K}}_{\mathrm{L}}{\mathrm{q}}_{\mathrm{m}}}+ \frac{1}{{\mathrm{q}}_{\mathrm{m}}}{\mathrm{C}}_{\mathrm{e}}$$

7 $${\mathrm{q}}_{\mathrm{e}}={\mathrm{B}}_{\mathrm{T}}{\mathrm{ln}(\mathrm{K}}_{\mathrm{F}})+{\mathrm{B}}_{\mathrm{T}}{\mathrm{lnC}}_{\mathrm{e}}$$

Ce (mg/L) is the diazinon concentration, qe (mg/g) is the adsorption capacity of the diazinon in optimal conditions, n and KF (mg/g) are Freundlich constants, AT is the equilibrium binding constant (L/mg) and B is the Temkin constant.

**Figure 12:**
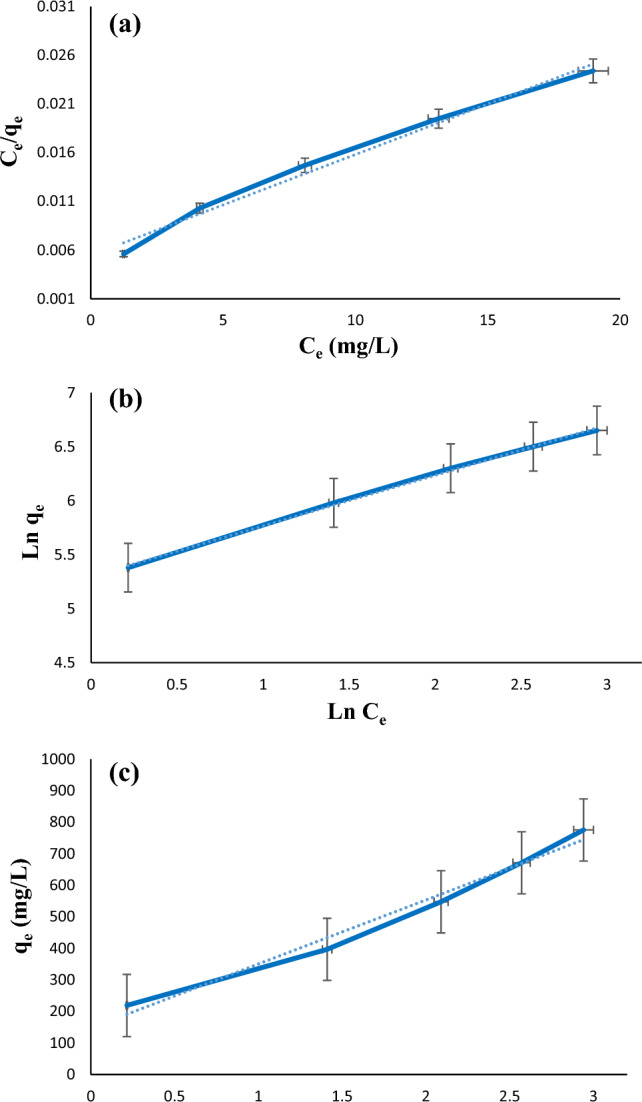
(**a**) Langmuir, (**b**) Freundlich, and (**c**) Temkin isotherms obtained for diazinon adsorption on LDH@TDI@THAM composite.

Freundlich, Langmuir, and Temkin isotherms for diazinon adsorption on LDH@TDI@THAM composite are compared in Fig. 12. It can be seen that Freundlich isotherm shows higher correlation coefficient (R^2^) than Langmuir and Temkin isotherms, which indicates that the diazinon adsorption on the LDH@TDI@THAM composite is more compatible with the Freundlich isotherm. Also, the maximum adsorption capacity at 40 °C was 1000 mg/g (Table 2).

**Table 2:** Parameters of Freundlich and Langmuir isotherms for diazinon adsorption on LDH@TDI@THAM composite.

Models	Parameters	LDH@TDI@THAM composite
Langmuir	q_max_ (mg/g)	1000
	K_L_	0.181
	R^2^	0.9859
Freundlich	K_f_	200.056
	n	2.13
	R^2^	0.9981
Temkin	R^2^	0.9811
	B	203.11
	A_T_	2.066

### Thermodynamic investigation

**Figure 13:**
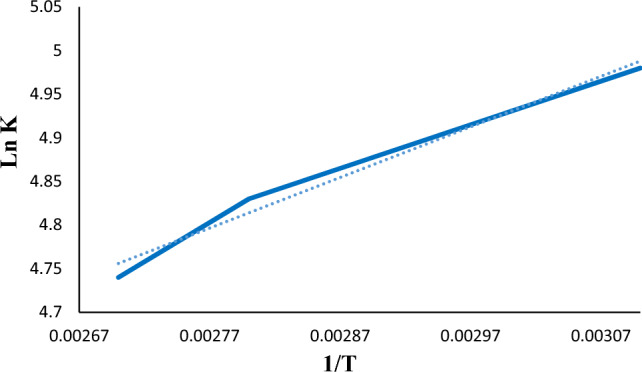
The plot of Van’t Hoff equation for diazinon adsorption.

The adsorption mechanism was further studied by calculating different thermodynamic parameters (ΔS°, ΔH°, and ΔG°) for diazinon adsorption on LDH@TDI@THAM composite using Eq. (7) and Eq. (8).

8 $${\mathrm{lnK}}_{\mathrm{d}}=\frac{\mathrm{\Delta S}}{\mathrm{R}}-\frac{\mathrm{\Delta H}}{\mathrm{RT}}$$

9 $$\mathrm{\Delta G}^\circ =\mathrm{\Delta H}^\circ -\mathrm{T \Delta S}^\circ$$

where T (K) is the temperature and R is the gas constant. Figure 13 and Table 3 briefly show the thermodynamic parameters related to diazinon adsorption in LDH@TDI@THAM composite. The obtained results clearly confirm the exothermicity of the diazinon adsorption process on the LDH@TDI@THAM composite (ΔH° = − 4.157 kJ/mol). This value for ΔH° confirms physical adsorption of diazinon on LDH@TDI@THAM composite. Also, the value obtained for ΔS° is indicative of high tendency of LDH@TDI@THAM composite for diazinon adsorption.

**Table 3:** Thermodynamic values for diazinon adsorption on LDH@TDI@THAM composite.

Adsorbent	ΔH° (kJ/mol)	ΔS° (J/mol-K)	ΔG° (kJ/mol)				
			298	313	338	353	368
LDH@TDI@THAM composite	− 4.15	− 28.51	− 3.88	− 3.87	− 3.84	− 3.83	− 3.81

### Adsorption mechanism

**Figure 14:**
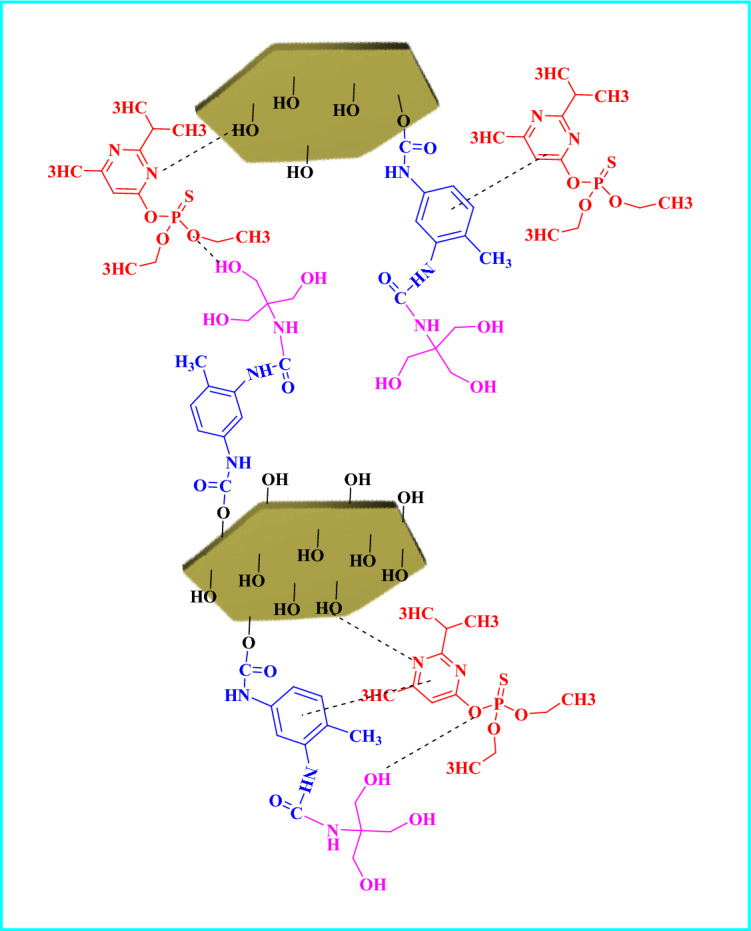
Schematic representation of diazinon adsorption mechanism on the LDH@TDI@THAM composite.

Figure 14 shows the absorption mechanism of diazinon pesticide removal using LDH@TDI@THAM composite. It is evident that the structure of the LDH@TDI@THAM composite contains three functional groups: OH, NH, and aromatic ring. Hence, the adsorption of diazinon may be due to hydrogen bonding between amine groups and electron-rich oxygen and electrostatic interaction. Also, the LDH@TDI@THAM composite contains benzene rings that can form π–π interactions with the benzene ring of diazinon.

### Recycling studies

**Figure 15:**
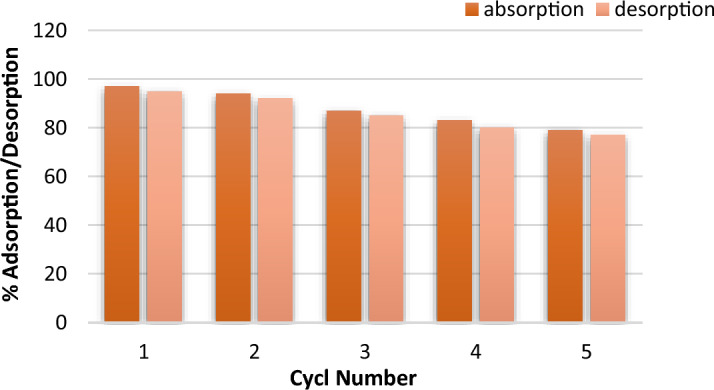
Adsorption–desorption isotherms obtained for diazinon adsorption on the LDH@TDI@THAM composite.

**Figure 16:**
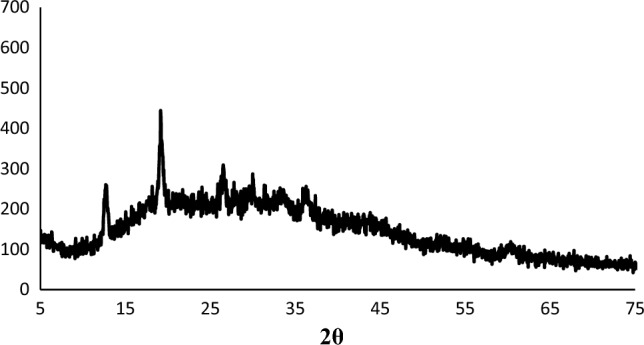
XRD patterns of reusability LDH@TDI@THAM composite.

**Figure 17:**
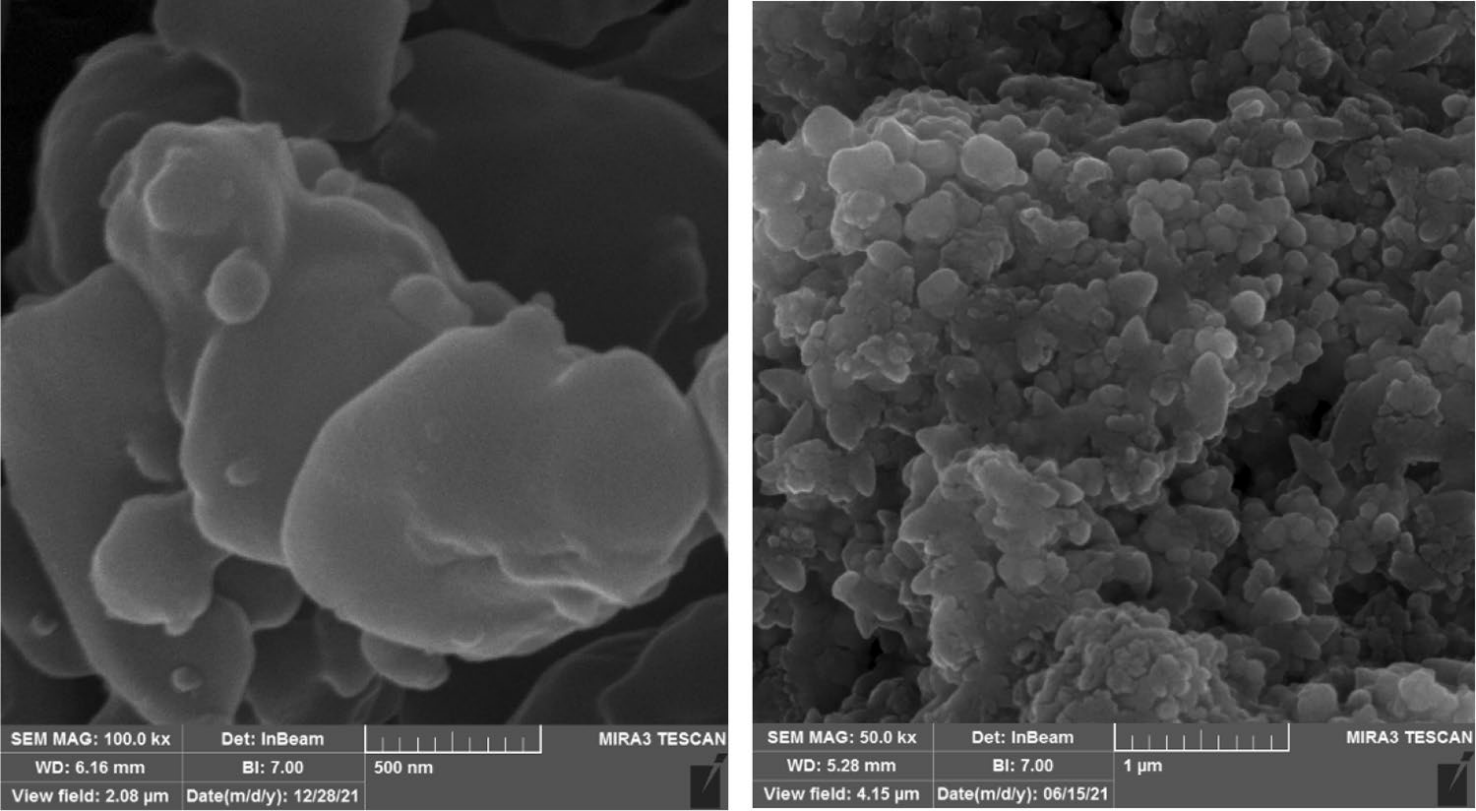
FESEM images of reusability LDH@TDI@THAM composites.

Reusability and stability for adsorbent materials during the adsorption process is an important factor. Thus, to investigate the reusability of LDH@TDI@THAM composite to remove diazinon in a mixture of NaCl (0.1 M) and HCl (0.1 M) as a detergent agent. For this reason, a mixture of NaCl (0.1 M) and HCl (0.1 M) was used to wash the LDH@TDI@THAM composite, followed by drying at 80 °C for 5 h. Therefore, according to Fig. 15, reusability of the LDH@TDI@THAM composite was investigated for four consecutive periods. XRD pattern and FESEM images of LDH@TDI@THAM composite after adsorption four periods (Figs. 16 and 17).

The diazinon adsorption capacity of LDH@TDI@THAM composite is compared in Table 4 with other adsorbents reported previously. The unique characteristics of LDH@TDI@THAM composite including simple preparation method, reusability, low cost, and high adsorption capacity have made this adsorbent superior to other adsorbents reported previously.

**Table 4:** compares the diazinon adsorption ability of LDH@TDI@THAM composite with other adsorbents reported previously.

Entry	Adsorbent	Adsorption capacity (mg/g)	References
1	NH_4_Cl-induced activated carbon	250	^1^
2	clay/GO/Fe_3_O_4_	7.38	^57^
3	Fe_3_O_4_-gg-montmorillonite	80	^36^
4	MNPs-AGENVC-CD	34.24	^58^
5	LDH@TDI@THAM composite	1000	This work
6	Zn–Al LDH	420	This work

## Experimental

### Materials and methods

All chemicals and solvents used were purchased from Aldrich or Merck. LDH@TDI@THAM composite was characterized by FT-IR (Shimadzu 8400 s), EDX (Numerix DXP-X10P), FESEM (TESCAN-MIRA3), and TGA (Bahr Company STA 504). X-ray diffraction (XRD) patterns of the composite were recorded on TW 1800 diffractometer (*λ*_CuKa_ = 1*.*54050 Å).

### General procedure for preparing Zn–Al LDH

LDH was prepared via urea-assisted coprecipitation procedure^59,60^.In a glass flask (200 mL), Zn(NO_3_)_2_.6H_2_O (2.56 g) and Al(NO_3_)_3_.9H_2_O (1.87 g) in aqueous urea solution (3 M, 100 mL) stirred at 100 °C for 12 h. Then, the temperature was reduced to 94 °C and kept in the aging mode for 12 h. Eventually, the prepared Zn–Al LDH was separated by centrifuging and then washed with deionized water to reach pH 7. Then it was dried at 80 °C for 24 h.

### Preparation of LDH@TDI@THAM composite

**Figure 18:**
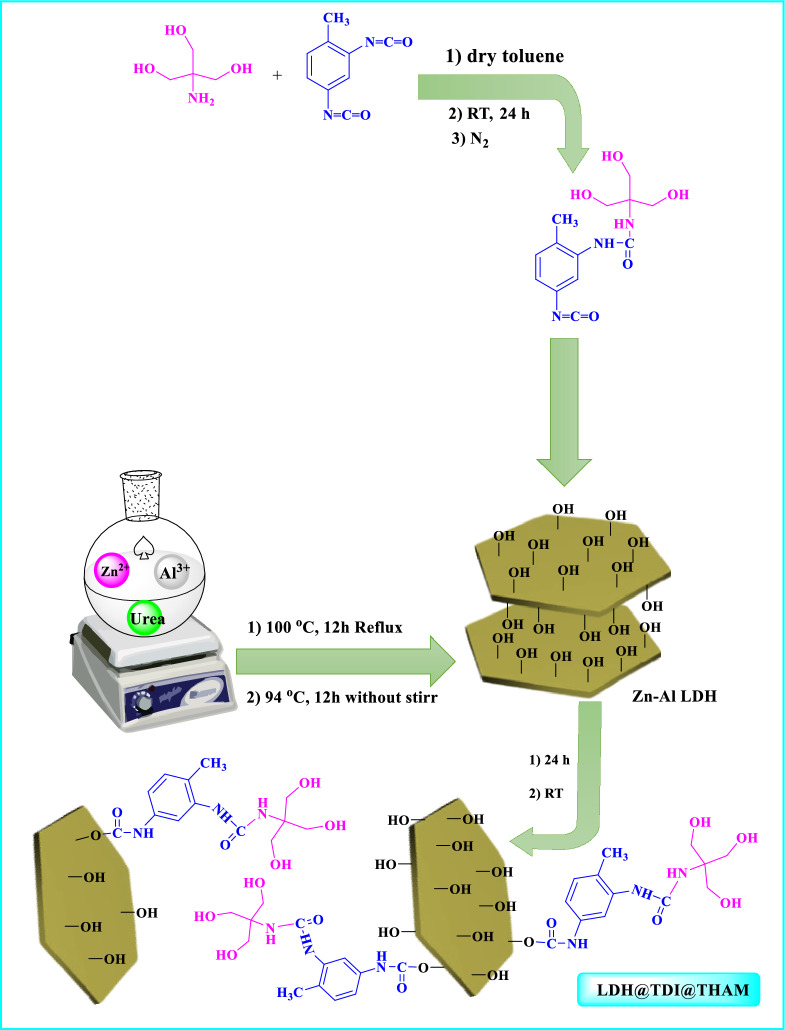
Preparation of LDH@TDI@THAM composite.

First, Tris(hydroxymethyl)aminomethane (THAM, 1 g) was dispersed in toluene (10 mL). Then2,4-toluene diisocyanate (TDI, 1.18 mL) was added and stirred at room temperature for 24 h under N_2_ atmosphere.In the following, Zn–Al LDH (0.5 g) was added and stirred at room temperature for another 24 h. Finally, the LDH@TDI@THAM composite was centrifuged, then washed with H_2_O and toluene, finally dried at 85 °C to 18 h (Fig. 18).

## Conclusions

In this work, LDH@TDI@THAM composite was prepared to remove diazinon from aqueous solutions. Also, the structure of the prepared adsorbent was investigated by different analyzes such as XRD, FTIR, EDX, TGA, and FESEM. LDH polymer composite as adsorbent showed a high affinity to absorb diazinon molecule. Moreover, kinetic studies have shown that diazinon adsorption on LDH@TDI@THAM composite fit the pseudo-second-order model. Also, the obtained data confirmed the suitability of the Freundlich isotherm model for diazinon adsorption by LDH@TDI@THAM composite. The maximum capacity obtained for LDH@TDI@THAM composite was 1000 mg/g. The thermodynamic data also confirmed the exothermic behavior of adsorption on LDH@TDI@THAM composite.

## Supplementary Information

Supplementary Information.

## Data Availability

All data generated or analyzed during this study are included in this published article [and its supplementary information files].
