# Supplementary material for: Preparation of novel Zn–Al layered double hydroxide composite as adsorbent for removal of organophosphorus insecticides from water
Source: Sci Rep. 2023 Jun 23;13:10215. doi: 10.1038/s41598-023-37070-8 (PMC10290108; doi:10.1038/s41598-023-37070-8)
Supplement: Supplementary file 1 — Supplementary Information. [file 41598_2023_37070_MOESM1_ESM.docx]

**Supporting information**

**Preparation of novel Zn-Al layered double hydroxide composite as adsorbent for removal of organophosphorus insecticides from water**

Nastaran Ghanbari, Hossein Ghafuri *

*Catalysts and Organic Synthesis Research Laboratory, Department of Chemistry, Iran University of Science and Technology, Tehran 16846‑13114, Iran*

** E-mail:* [*ghafuri@iust.ac.ir*](mailto:ghafuri@iust.ac.ir)

**Experimental**

**Materials and methods**

All chemicals and solvents used were purchased from Aldrich or Merck. LDH@TDI@THAM composite was characterized by FT-IR (Shimadzu 8400 s), EDX (Numerix DXP-X10P), FESEM (TESCAN-MIRA3), and TGA (Bahr Company STA 504). X-ray diffraction (XRD) patterns of the composite were recorded on TW 1800 diffractometer (*λ*_CuKa_ =1*.*54050 Å).

**General procedure for preparing Zn-Al LDH**

LDH was prepared via urea-assisted coprecipitation procedure ^78^.In a glass flask (200 mL), Zn(NO_3_)_2_.6H_2_O (2.56 g) and Al(NO_3_)_3_.9H_2_O (1.87 g) in aqueous urea solution (3 M, 100 mL) stirred at 100 °C for 12 h. Then, the temperature was reduced to 94°C and kept in the aging mode for 12 h. Eventually, the prepared Zn-Al LDH was separated by centrifuging and then washed with deionized water to reach pH 7. Then it was dried at 80°C for 24 h.

**Preparation of LDH@TDI@THAM composite**

First, Tris(hydroxymethyl)aminomethane (THAM, 1 g) was dispersed in toluene (10 mL). Then2,4-toluene diisocyanate (TDI, 1.18 mL) was added and stirred at room temperature for 24 h under N_2_ atmosphere.In the following, Zn-Al LDH (0.5 g) was added and stirred at room temperature for another 24 h. Finally, the LDH@TDI@THAM composite was centrifuged, then washed with H_2_O and toluene, finally dried at 85°C to 18 h.
